# The Effects of Guided Imagery on Patients Being Weaned from Mechanical Ventilation

**DOI:** 10.1155/2015/802865

**Published:** 2015-11-10

**Authors:** LeeAnna Spiva, Patricia L. Hart, Erin Gallagher, Frank McVay, Melida Garcia, Karen Malley, Marsha Kadner, Angela Segars, Betsy Brakovich, Sonja Y. Horton, Novlette Smith

**Affiliations:** ^1^WellStar Health System, Center for Nursing Excellence, WellStar Development Center, 2000 South Park Place, Atlanta, GA 30339, USA; ^2^Kennesaw State University, 1000 Chastain Road, Prillaman Hall Building 41, Kennesaw, GA 30144, USA; ^3^WellStar Health System, Center for Nursing Excellence, 2000 South Park Place, Atlanta, GA 30339, USA; ^4^WellStar Cobb Hospital, 3950 Austell Road, Austell, GA 30106, USA; ^5^WellStar Kennestone Hospital, 677 Church Street, Marietta, GA 30060, USA; ^6^WellStar Windy Hill Hospital, 2540 Windy Hill Road, Marietta, GA 30067, USA

## Abstract

The study purpose was to assess the effects of guided imagery on sedation levels, sedative and analgesic volume consumption, and physiological responses of patients being weaned from mechanical ventilation. Forty-two patients were selected from two community acute care hospitals. One hospital served as the comparison group and provided routine care (no intervention) while the other hospital provided the guided imagery intervention. The intervention included two sessions, each lasting 60 minutes, offered during morning weaning trials from mechanical ventilation. Measurements were recorded in groups at baseline and 30- and 60-minute intervals and included vital signs and Richmond Agitation-Sedation Scale (RASS) score. Sedative and analgesic medication volume consumption were recorded 24 hours prior to and after the intervention. The guided imagery group had significantly improved RASS scores and reduced sedative and analgesic volume consumption. During the second session, oxygen saturation levels significantly improved compared to the comparison group. Guided imagery group had 4.88 less days requiring mechanical ventilation and 1.4 reduction in hospital length of stay compared to the comparison group. Guided imagery may be complementary and alternative medicine (CAM) intervention to provide during mechanical ventilation weaning trials.

## 1. Introduction

Mechanical ventilation is a life-sustaining treatment for respiratory compromised patients by reducing the work to breathe, oxygenate tissue, and eliminate carbon dioxide [1]. It is a costly treatment estimated at $27 billion a year representing 12% of hospital costs [2]. Therefore, early assessment of weaning readiness and implementation of standardized weaning trials to transition patients from full ventilator support to spontaneous breathing with the goal of early extubation [3, 4] are essential to prevent unintended consequences from prolonged ventilation and weaning [5]. Patients requiring mechanical ventilation may experience unintended consequences including but not limited to anxiety and inability to relax [6, 7], psychological and emotional distress at being unable to communicate [8], and delusional memories [7]. Analgesic and sedatives are commonly administered to reduce these symptoms. Consequently, pharmacologic interventions including sedatives and analgesics have notable side effects and are found to prolong mechanical ventilation and contribute to a higher hospital and/or intensive care unit (ICU) length of stay [9–11]. As mechanical ventilation duration increases, risk of hospital-acquired complications increases contributing to higher mortality and morbidity rates [2, 12–14].

Further work is needed to explore the effects of guided imagery, a complementary and alternative medicine (CAM) technique, used to optimize a mind-body connection. Guided imagery is used to focus on pleasant mental images to promote healing and relaxation, manage symptoms, and ultimately contribute to critically ill patients' well-being [15–18]. Guided imagery may have the potential to reduce the frequency and severity of symptoms in patients weaning from mechanical ventilation. Guided imagery may assist with shifting focus away from the weaning trial to acquiring a level of relaxation. Therefore, we conducted a study to determine the effects of guided imagery in mechanically ventilated patients undergoing active ventilator weaning on sedation levels, sedative and analgesic volume consumption, and physiological responses. Additionally, we assessed nurse perception of the feasibility and satisfaction of using guided imagery as an intervention.

Guided imagery has been used in a variety of patient populations including preoperative patients [19], antepartum patients [20], community-dwelling older adults [21], patients with cancer [22, 23], cardiac patients [24–26], and patients with chronic pain [27, 28]. Patients recovering from same day head and neck surgery had a significant reduction in anxiety and pain levels and postanesthesia care unit (PACU) length of stay was nine minutes less compared to the control group [19]. There is evidence that guided imagery is an effective intervention to reduce maternal stress, fatigue, and anxiety for pregnant African American women in the second trimester [20]. Guided imagery was shown to improve self-reported leisure time behavior, reduce mobility test time, and reduce the fear of falling in older adults [21].

Researchers found lower respiratory and heart rates and blood pressure measurements and felt the sessions were beneficial for patients undergoing radiation therapy for breast cancer [23]. Similarly, thyroid patients undergoing radioactive iodine therapy had reduced fatigue and stress levels [22].

Guided imagery has been used as an intervention with cardiac patients including post-open heart surgery patients [24, 26] and has shown to reduce length of stay, anxiety, and pain levels. In addition, percutaneous transluminal coronary angiography patients [25] had reduced anxiety and pain levels and improved heart and respiratory rates and blood pressure after listening to a guided imagery cassette for 18 minutes.

In addition, guided imagery has been shown to reduce musculoskeletal pain and medication usage, including analgesics, in osteoarthritis patients from baseline to four months [27]. Similarly, patients with fibromyalgia who received guided imagery as an intervention had lower pain and depression levels compared to usual care [28]. However, two systematic reviews concluded that guided imagery used for musculoskeletal pain [29] and nonmusculoskeletal pain [30] were inconclusive due to lack of methodological rigor. Furthermore, the beneficial effects of guided imagery have not been studied as an intervention to provide to patients weaning from mechanical ventilation. Therefore, the specific aims of this quasi-experimental, repeated measure with intervention and comparison groups study were to assess the effects of guided imagery on patients being weaned from mechanical ventilation.

## 2. Methods

### 2.1. Participants

Patients were recruited from an integrated healthcare system that included two community acute care hospitals with seven ICUs located in the southeastern United States from August 1, 2012, to March 10, 2014. One hospital served as the comparison group and provided routine care (no intervention), while the other hospital provided the guided imagery intervention. The two hospitals were chosen based on similar patient population (age, reason for ICU admission, severity of illness, etc.), and evidence-based mechanical ventilation order set was followed for daily spontaneous breathing trials used to decrease practice variation. Additionally, the four researchers conducting the intervention were based at the hospital receiving the intervention. Patient inclusion criteria were (a) age greater than 18 years, (b) actively weaning from mechanical ventilation (the process of gradual reduction of ventilator support) per the hospital's standard weaning criteria, and (c) no hearing impairment. Nurse inclusion criteria included directly caring for a patient receiving the intervention. With a power of .80, an alpha value of .05, and a medium effect of 0.25, 34 participants were needed for the study [31, 32].

The study was reviewed and approved by Kennesaw State University Institutional Review Board and the study site's nursing research council. Informed consent was obtained at the beginning of the study by one of the study researchers from each patient's surrogate due to the patient consumption of sedatives and analgesics. In order to protect confidentiality, each participant was assigned a unique identifier.

### 2.2. Intervention

If patient was receiving continuous infusions of sedation and/or analgesic, the infusions were stopped in order to assess patient readiness for weaning and extubation. Prior to the intervention, patient's sedation level was assessed before weaning to ensure patient was rested, comfortable, and not lethargic, when weaning started. For patients enrolled in the guided imagery group, two separate sessions were held on two consecutive days, each lasting 60 minutes and offered during morning weaning trials. The structured, guided imagery, produced by Guided Imagery, Inc., was delivered via PLAYWAY device, 5 × 7 inches' plastic case. The case included a four-track preloaded 60-minute audio book that required AAA battery. Disposable earphones were connected to the device. The guided imagery was narrated with a faint, soft voice, instructing the patient to relax. The session started approximately 20 minutes prior to weaning. Patients listened to the content for 60 minutes during the spontaneous breathing trial from mechanical ventilation. Four study researchers delivered the intervention and remained with the patient throughout the session. The length of time of the intervention was based on the hospital's average duration of a weaning trial (30 to 120 minutes); and the period of the delivery of the intervention was based on the weaning process occurring each morning. For patients enrolled in the comparison group, the intervention did not occur and routine patient care management was in accordance with institutional standards. Routine monitoring included pulse oximetry, five-lead electrocardiography, heart rate, respiratory rate, and blood pressure measurements.

### 2.3. Measurement

The Richmond Agitation-Sedation Scale (RASS) was developed to titrate sedation and pain control [33]. The 10-point scale ranges from unresponsive (−5) to calm and alert (0) to combative (+4). The RASS scale has undergone extensive reliability and validity testing and is sensitive to detect changes in sedation status against level of consciousness and delirium and correlated with sedative and analgesic medication doses [34]. The RASS score is used to titrate sedation and pain control for ICU patients at the study hospitals [33, 34].

The Acute Physiology and Chronic Health Evaluation (APACHE II) provided an estimate of illness severity and in-hospital mortality of ICU patients. Twelve variables are used to calculate APACHE II score. Extensive reliability and validity testing has been conducted on APACHE II. The researchers conducted a retrospective chart review and recorded the worst APACHE II score during the initial 24 hours of the ICU stay [35].

The researchers developed a survey including four questions addressing feasibility and satisfaction of using guided imagery as an intervention. The survey is rated on a 5-point Likert scale (1 =* strongly disagree* and 5 =* strongly agree*). Nurses directly involved with patients receiving the intervention completed the surveys immediately after the intervention session. Completion of the survey by the nurse served as his or her consent to participate.

### 2.4. Procedures

Several times each week, the researchers communicated with the ICUs to identify potential study participants. All eligible patients were enrolled if patient met the study's inclusion criteria. Measurements were recorded by the study researchers at baseline and 30- and 60-minute intervals and included heart rate, systolic and diastolic blood pressure, respiratory rate, oxygen saturation, and RASS scores. Vital signs were measured indirectly from the noninvasive module on the monitor. Prior to data collection, the bedside monitors were tested and calibrated by the bioengineering department. Total amounts (volume) of continuous intravenous sedatives and analgesics administered in a 24-hour timeframe were converted into milliliters and recorded from the electronic documentation system by the study researchers. Sedative and analgesic amounts were evaluated during a continuous 24-hour period before and after the intervention. Commonly administered sedatives included Diprivan (propofol), dexmedetomidine (Precedex), midazolam (Versed), and lorazapam (Ativan). Commonly administered analgesics included fentanyl and morphine. The researchers reviewed the patient's medical record to collect demographic data and data to calculate APACHE II.

### 2.5. Data Analysis

Data were analyzed using SPSS 22.0 software for Windows (SPSS, Inc., IBM Company, Armonk, NY, USA). An independent *t*-test, Chi-square test, and Mann-Whitney *U* test were conducted to examine if any differences existed between the comparison and intervention group. A Friedman test was conducted to determine changes over time with the RASS scores and sedative volumes followed by a post hoc analysis with Wilcoxon signed-rank test with a Bonferroni correction applied. One-way repeated measures analysis of variance (ANOVA) was conducted to test the effect of guided imagery on critically ill ventilated patients' and physiological responses during both sessions at baseline, 30 minutes, and 60 minutes. To detect differences with analgesic use, *t*-tests were conducted. Significance level was set at *P* < .05. Post hoc tests were conducted to determine where the difference in means occurred.

## 3. Results

### 3.1. Sample

**Figure 1:**
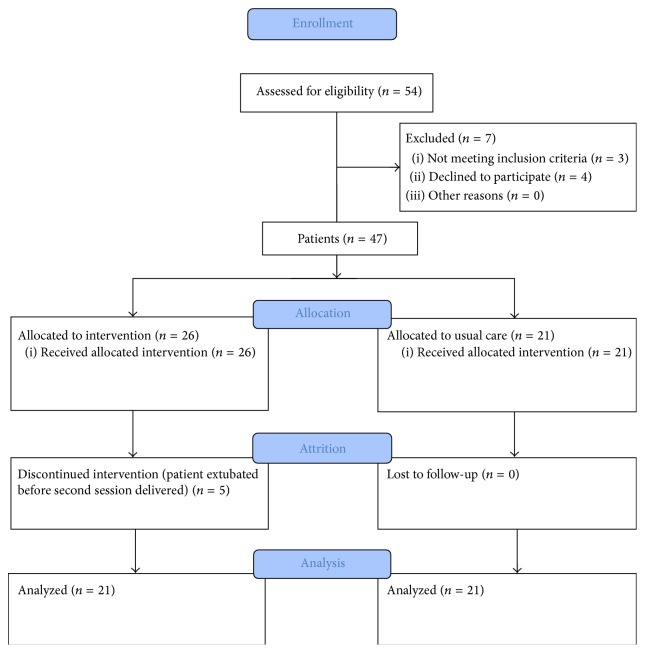
Flow diagram of patient enrollment. Adapted from Schulz K. F., Altman D. G., and Moher D. (2010). For the CONSORT Group.

Sample demographic characteristics are presented in Table 1. All variables between the groups were normally distributed except gender, race, RASS scores, and sedation volume. Initially, 54 patients were screened, and 42 patients receiving mechanical ventilation supported via oral endotracheal tube met study criteria and participated (Figure 1). Twenty-one patients received two 60-minute guided imagery sessions (intervention). The first session occurred within 24 hours of initial intubation and the second session followed 48 hours later. Another 21 patients served as the comparison group with no intervention and only data collection occurred within 24 hours of intubation and 48 hours later. The majority were white (69%) females (54.8%) with a mean age of 64.6 (SD, 13.25). Most patients were being treated with assist control (66.7%) and primary reasons for ICU admission included respiratory (59.5%), cardiac (28.6%), or other (11.9%) reasons. All patients who received the intervention were receiving one or more continuous intravenous sedative and/or analgesic infusions compared to only 16 patients in the comparison group (*χ* ^2^ = 5.76; *P* = .02). The APACHE mean score was 24.36 (SD, 7.42).

### 3.2. Sedation and Analgesics

The most significant effects of the intervention included improved RASS scores and a decrease in sedative and analgesic volume consumption (Table 2). During the first (*χ* ^2^(2) = 17.45, *P* = .000) and second (*χ* ^2^(2) = 7.65, *P* = .022) sessions there was a statistically significant difference in the RASS scores over the three time points. For the first-session median (IQR) baseline and 30-minute and 60-minute RASS scores were −1.00 (−2.00 to 0), −1.00 (−1.25 to 0), and −1.00 (−2.00 to 0), respectively. There were significant differences between first-session baselines and 30-minute RASS scores (*Z* = −3.380, *P* = .001) and baseline and 60-minute RASS scores (*Z* = −3.252, *P* = .001). The second-session median IQR baseline and 30-minute and 60-minute RASS scores were −1.00 (−2.00 to 0), 0 (−1.00 to 0), and 0 (−1.00 to 0), respectively. There were significant differences between first-session baselines and 30-minute RASS scores (*Z* = −2.524, *P* = .012) and baseline and 60-minute RASS scores (*Z* = −2.480, *P* = .013). As shown in Table 2, over time the intervention group's RASS scores decreased significantly from baseline (M = −2.10) to 30 minutes (M = −1.57; M  Difference = −.53; *P* = .01) and from baseline to 60 minutes (M = −1.19; M  Difference = −.91; *P* = .00). Additionally, a significant decrease in RASS scores was noted (M  Difference = −.38; *P* = .02) between the 30-minute interval (M = −1.57) and the 60-minute interval (M = 1.19). During the second session, the intervention group's RASS score decreased from baseline (M = −1.67) to 30 minutes (M = −1.08; M  Difference = −.59) and from baseline to 60 minutes (M = 0, M  Difference = −1.67).

There was a statistically significant difference in the sedative volumes over the four time points (*χ* ^2^(3) = 9.90, *P* = .019). The median (IQR) 24 hours prior to (session 1), 24 hours after (session 1), 24 hours prior to (session 2), and 24 hours after (session 2) cumulative sedative volume totals were 145 (10 to 232), 42 (0 to 152.65), 3 (0 to 208), and 0.5 (0 to 115.17), respectively. There were significant differences between first sessions' prior and after 24-hour cumulative volume sedative totals (*Z* = −3.009, *P* = .003) and first sessions' prior and session 2 after (*Z* = −2.633, *P* = .008). The intervention group had a significant reduction in sedative volumes (24 hours' cumulative amount) before and after the first and second intervention sessions. The 24-hour cumulative volume was reduced by 140.06 mL. Additionally, the intervention group had a significant reduction in analgesic volumes before and after the first intervention session (*t*_20_ = 2.77; *P* = .01) and a decrease during the second intervention but not significant (Table 2).

### 3.3. Physiological Responses

The intervention group mean heart rate, respiratory rate, and oxygen saturation remained well below the comparison group (Table 3). For the first intervention session only, heart rate differed significantly over the three time periods (*F*_2,80_ = 3.91; *P* = .02). Respiratory rate differed significantly over time during sessions one (*F*_2,80_ = 4.45; *P* = .02) and two (*F*_2,60_ = 3.02; *P* = .05). Both groups' heart and respiratory rates increased from baseline to 60 minutes; however, the intervention group had a lower heart and respiratory rate compared to the comparison group over the three time intervals. During the second intervention, there was a significant difference between the two groups' oxygen saturation levels (*F*_2,60_ = 3.11; *P* = .05). The intervention group had higher oxygen saturation levels during all three time periods compared to the comparison group. Furthermore, the guided imagery group had 4.88 less mechanical ventilation days compared to the comparison group (*t*_39_ = 1.33; *P* = .193). The guided imagery group hospital length of stay was 1.4 less days compared to the comparison group (*t*_40_ = .33; *P* = .74).

### 3.4. Staff Perception

Of the 42 surveys that were distributed, 23 nurses (55%) completed the survey with mean scores for each question ranging from 4.09 to 4.83. Nurses felt that guided imagery was an effective nursing intervention (M = 4.83, SD = .39). Nurses felt that the intervention was successfully incorporated into the weaning process (M = 4.09, SD = .95) and simple to implement (M = 4.13, SD = 1.0) and the intervention met the intended purpose (M = 4.09, SD = 1.08).

## 4. Discussion

To date, research conducted has focused on deployment of interventions with no research identified using guided imagery as an intervention in patients who are being actively weaned from the ventilator. This study is unique in using guided imagery as an intervention in mechanically ventilated patients who were being actively weaned from the ventilator. Despite the intervention group having higher RASS scores and receiving continuous sedative and analgesic infusions, we demonstrated improved RASS scores, reduced sedative and analgesic volume consumption, and higher oxygen saturation levels. Furthermore, we found that patients who received the intervention had a shorter time on the ventilator and shorter length of stay. We were able to demonstrate a significant improvement in actual sedative and analgesic volume intake in relation to using guided imagery as an intervention despite 24% of the interventions groups' baseline RASS score being greater than or equal to minus four (−4) indicating that the patient was deeply sedated.

Throughout the intervention, heart rate, diastolic blood pressure, and oxygen saturation levels remained within normal range. Similar to other researcher findings that used guided imagery, we found that heart and respiratory rates were significantly lower over time for the intervention group compared to the comparison group [23, 25]. The comparison group's respiratory rate increased and oxygen consumption declined. We did not find significant improvements in blood pressure but other medications including cardiac medications could have masked the intervention effects. Similar to Deisch et al. [24] and Halpin et al. [26] patients who received the guided imagery intervention had reduced length of hospital stay (1.4 less days) and 4.88 less mechanical ventilation days compared to the comparison group.

Complementary and alternative medicine therapy such as guided imagery may be a part of the multimodal treatment approach and serve as a substitute to administering high doses of sedatives to assist with keeping the patient calm and relaxed. Nurses perceived the intervention as effective and easily incorporated into the weaning process.

Our study had several limitations. The sample was primarily white females admitted to ICU with a respiratory problem. Sedation levels and sedative and analgesic use and practices may have varied between the hospitals and affected measurements. We only looked at volumes of sedatives and analgesics, as most of these medications are weight based; comparing volume of medications infused between groups is a limitation. Additionally, before intervention the intervention group had higher cumulative amounts of sedative and analgesics that might have influenced the amount of sedatives and analgesics needed during and after the intervention. Both hospitals' ventilator weaning is assumed by the respiratory therapist guided by standardized protocols. Daily weaning occurred in the mornings and intervention effects may have been different later in the day. Certain medications may have masked the intervention effects as we did not control for prescribed medications such as cardiac medications. Secondary to one hospital serving as the intervention hospital and the other serving as the control hospital, any hospital effect is potentially confounded by the intervention effect. By carrying out the intervention at one hospital only, the researchers were hoping to lessen the threat of treatment diffusion. It is difficult to be blinded to the intervention when the researchers had to deliver the intervention to the patient. A randomized controlled trial (RCT) was not conducted secondary to conditions that either occurred daily and/or were planned in the ICU that the researchers had no control over things including but not limited to noise levels, patient volumes, ICU renovations, and transition from one electronic medical record to another which would have added to additional study limitations. It is suggested to replicate the study randomizing the intervention at both hospitals to see if the findings of the present study are generalizable.

As we noted, weaning trials and intervention sessions occurred during the morning hours. We attempted to obtain surrogates' perceptions of ventilated patients who listened to the guided imagery. We did not capture enough data for analysis primarily due to low participation. Typically, the patients' surrogate consented for the patient to participate the day prior to the actual intervention and/or the surrogate was not present during the entire weaning process and intervention not meeting study criteria. Future investigators may want to involve patients' surrogate in intervention and weaning process to promote patient- and family-centered care. In addition, patients' surrogates were not always present at the hospital with the patient and initial weaning trial was unpredictable which at times made recruitment and data collection a challenge for the researchers. ICU nurses' perception of guided imagery as an effective intervention to implement was rather high; however, the four researchers delivering the intervention may have inadvertently positively skewed the nurses' perception of guided imagery. Furthermore, the intervention effect sustained beyond the study time is unknown.

## 5. Conclusions

Guided imagery may be a CAM intervention to provide during mechanical ventilation weaning trials. Guided imagery appeared to be effective, safe, and feasible intervention to use in patients being weaned from mechanical ventilation. Future research is needed including a larger randomized controlled trial examining the effect of guided imagery use with a larger sample with a longer tracking period in relation to patient outcomes.

## Figures and Tables

**Table 1:** Demographic characteristics.

Characteristic	Comparison group (*n* = 21)	Guided Imagery (*n* = 21)	Total (*N* = 42)	*P*
Age, y				
Mean (SD)	64 (14.1)	65.2 (12.7)	64.60 (13.25)	.78
Median, range	61, 35–93	66, 39–89	64.50, 35–93	
Female sex, %	15, 71.4%	8, 38.1%	23, 54.8%	.03
Race, %				.05
White	12, 57.1%	17, 81%	29, 69%	
African American	7, 33.3%	1, 4.8%	8, 19%	
Others	2, 9.5%	3, 14.3%	5, 11.9%	
Marital status, %				.87
Married	6, 28.6%	14, 66.7%	20, 47.6%	
Single	6, 28.6%	2, 9.55	8, 19%	
Others	9, 42.9%	5, 23.8%	14, 33.3%	
Number of comorbidities				
Mean (SD)	5.9 (2.8)	4.7 (2.6)	5.29 (2.7)	.14
Median, range	6, 1–15	4, 1–10	5, 1–15	
Acute physiology and chronic health evaluation (APACHE) score				
Mean (SD)	22.9 (8)	25.8 (6.7)	24.36 (7.42)	.21
Median, range	24, 10–39	24, 9–38	24, 9–39	
Primary reason for ICU admission, %				.09
Respiratory	14, 66.7%	11, 52.4%	25, 59.5%	
Cardiac	4, 19%	8, 38.1%	12, 28.6%	
Others	3, 14.3%	2, 9.5%	5, 11.9%	
Hospital length of stay				
Mean (SD)	21.33 (15.9)	19.90 (11.1)	20.63 (13.6)	.74
Total days on ventilator				
Mean (SD)	13.14 (15.2)	8.26 (7.3)	10.7 (12.1)	.20
Median, range	7, 2–56	7, 1–29	7, 1–56	
Ventilator mode				.06
Assist control, *n* %	18, 85.7%	10, 47.6%	28, 66.7%	
Synchronized intermittent mandatory, *n* %	2, 9.5%	3, 14.3%	5, 11.9%	
Pressure control, *n* %	1, 4.8%	8, 38.1%	9, 21.4%	

**Table 2:** Sedation levels, sedative, and analgesic outcomes.

Characteristic	Comparison group (*n* = 21), mean (SD)	Guided imagery (*n* = 21), mean (SD)	*P*
*Session I*			
Baseline RASS score	−.38 (1.1)	−2.10 (1.4)	.000
30-minute RASS score	0.1 (1.2)	−1.57 (1.4)	.000
60-minute RASS score	−.29 (1.2)	−1.19 (1.8)	.086
24-hour cumulative amount (mL) of sedative infused before	133.18 (143.28) (16 doses)	218.66 (243.62) (19 doses)	.284
24-hour cumulative amount (mL) of analgesic infused before	48.92 (83.77) (11 doses)	95.28 (128.12) (14 doses)	.173
24-hour cumulative amount (mL) of sedative infused after	111.77 (156.24) (13 doses)	78.59 (93.61) (13 doses)	.737
24-hour cumulative amount (mL) of analgesic infused after	54.69 (89.28) (11 doses)	18.10 (51.36) (8 doses)	.111
*Session II*			
Baseline RASS score	−.55 (.8)	−1.67 (2)	.043
30-minute RASS score	−.35 (.9)	−1.08 (1.8)	.162
60-minute RASS score	−0.55 (.8)	0 (1.6)	.181
24-hour cumulative amount (mL) of sedative infused before	109.24 (146.74) (14 doses)	166.54 (259.61) (10 doses)	.803
24-hour cumulative amount (mL) of analgesic infused before	24 (52.67) (6 doses)	2.52 (9.13) (4 doses)	.073
24-hour cumulative amount (mL) of sedative infused after	125.93 (188.45) (15 doses)	38.13 (75.78) (3 doses)	.024
24-hour cumulative amount (mL) of analgesic infused after	21.14 (56.36) (4 doses)	.49 (1.53) (3 doses)	.101

**Table 3:** Physiological outcomes.

Characteristic	Comparison group (*n* = 21)	Guided imagery (*n* = 21)	Total (*N* = 42)	*P*
*Session I*				
Baseline, mean (SD)				
Heart rate	84.7 (16.8)	79.1 (19)	81.9 (17.7)	.317
Systolic blood pressure (BP)	124.7 (22.9)	126.6 (19)	125.6 (21)	.777
Diastolic BP	59.3 (11.8)	64.1 (17.4)	61.7 (17.9)	.305
Respiratory rate	21.8 (4.5)	19.8 (4.8)	20.8 (4.7)	.170
Oxygen saturation	97.6 (2.5)	97.6 (2.4)	97.6 (2.4)	1.000
30 minutes, mean (SD)				
Heart rate	89.3 (22.2)	81.6 (18)	85.5 (20.3)	.220
Systolic BP	133.9 (17.4)	127.2 (20)	130.5 (19)	.258
Diastolic BP	64.5 (12.2)	64.7 (16.1)	64.6 (14.1)	.966
Respiratory rate	22.6 (4)	20.3 (6.8)	21.5 (5.7)	.186
Oxygen saturation	97.1 (2.7)	97.2 (3)	97.1 (2.8)	.915
60 minutes, mean (SD)				
Heart rate	90.3 (16.4)	82.7 (16)	86.5 (16.3)	.130
Systolic BP	129.6 (24.4)	130.2 (25)	129.9 (24.3)	.940
Diastolic BP	62 (11.9)	63.8 (20)	62.9 (16.2)	.728
Respiratory rate	24.6 (4.7)	21.6 (8.1)	23.1 (6.7)	.148
Oxygen saturation	97.2 (2.5)	97.1 (2.8)	97.1 (2.6)	.818
*Session II*				
Baseline, mean (SD)				
Heart rate	86.5 (17.4)	77.4 (14.8)	83.1 (16.8)	.141
Systolic BP	123.8 (25.8)	133.9 (20)	127.6 (24.1)	.254
Diastolic BP	60 (12.3)	65.8 (21.3)	62.2 (16.2)	.338
Respiratory rate	23.4 (3.6)	20.9 (4.6)	22.5 (4.1)	.099
Oxygen saturation	96.6 (3)	96.9 (2.2)	96.7 (2.7)	.733
30 minutes, mean (SD)				
Heart rate	94 (14.7)	82.8 (19.4)	89.8 (17.2)	.073
Systolic BP	141.4 (25.8)	135.8 (13.7)	139.3 (22)	.622
Diastolic BP	63.3 (10.8)	66.7 (17.7)	64.6 (13.6)	.426
Respiratory rate	22.2 (4.8)	23.8 (7)	22.8 (5.7)	.507
Oxygen saturation	96.9 (2.8)	97.2 (2.3)	97 (2.6)	.427
60 minutes, mean (SD)				
Heart rate	92.4 (21.2)	81.5 (12.5)	88.3 (19)	.119
Systolic BP	124.6 (21.7)	136.9 (21.7)	129.2 (22.2)	.130
Diastolic BP	62.5 (14.9)	68.7 (15.6)	64.8 (15.2)	.271
Respiratory rate	23.3 (5.5)	20.8 (5.6)	22.4 (5.6)	.235
Oxygen saturation	95.6 (3.8)	97.9 (1.6)	96.5 (3.3)	.024
